# Inflammatory plasma biomarkers in subjects with preclinical Alzheimer’s disease

**DOI:** 10.1186/s13195-022-01051-2

**Published:** 2022-08-03

**Authors:** Samantha Prins, Marieke L. de Kam, Charlotte E. Teunissen, Geert Jan Groeneveld

**Affiliations:** 1 Centre for Human Drug Research, Leiden, the Netherlands; 2 Leiden University Medical Center, Leiden, the Netherlands; 3 Neurochemistry Laboratory, Department of Clinical Chemistry, Amsterdam Neuroscience, Amsterdam UMC, Vrije Universiteit Amsterdam, Amsterdam, the Netherlands

**Keywords:** Preclinical Alzheimer’s disease, Neuroinflammation, GFAP, YKL-40, MCP-1, Eotaxin-1

## Abstract

**Background:**

This study investigated plasma biomarkers for neuroinflammation associated with Alzheimer’s disease (AD) in subjects with preclinical AD compared to healthy elderly. How these biomarkers behave in patients with AD, compared to healthy elderly is well known, but determining these in subjects with preclinical AD is not and will add information related to the onset of AD. When found to be different in preclinical AD, these inflammatory biomarkers may be used to select preclinical AD subjects who are most likely to develop AD, to participate in clinical trials with new disease-modifying drugs.

**Methods:**

Healthy elderly (*n*= 50; age 71.9; MMSE >24) and subjects with preclinical AD (*n*=50; age 73.4; MMSE >24) defined by CSF Aβ1-42 levels < 1000 pg/mL were included. Four neuroinflammatory biomarkers were determined in plasma, GFAP, YKL-40, MCP-1, and eotaxin-1. Differences in biomarker outcomes were compared using ANCOVA. Subject characteristics age, gender, and APOE ε4 status were reported per group and were covariates in the ANCOVA. Least square means were calculated for all 4 inflammatory biomarkers using both the Aβ+/Aβ− cutoff and Ptau/Aβ1-42 ratio.

**Results:**

The mean (standard deviation, SD) age of the subjects (*n*=100) was 72.6 (4.6) years old with 62 male and 38 female subjects. Mean (SD) overall MMSE score was 28.7 (0.49) and 32 subjects were APOE ε4 carriers. The number of subjects in the different APOE ε4 status categories differed significantly between the Aβ+ and Aβ− groups. Plasma GFAP concentration was significantly higher in the Aβ+ group compared to the Aβ− group with significant covariates age and sex, variables that also correlated significantly with GFAP.

**Conclusion:**

GFAP was significantly higher in subjects with preclinical AD compared to healthy elderly which agrees with previous studies. When defining preclinical AD based on the Ptau181/Aβ1-42 ratio, YKL-40 was also significantly different between groups. This could indicate that GFAP and YKL-40 are more sensitive markers of the inflammatory process in response to the Aβ misfolding and aggregation that is ongoing as indicated by the lowered Aβ1-42 levels in the CSF. Characterizing subjects with preclinical AD using neuroinflammatory biomarkers is important for subject selection in new disease-modifying clinical trials.

**Trial registration:**

ISRCTN.org identifier: ISRCTN79036545 (retrospectively registered).

## Background

Biomarkers for Alzheimer’s disease (AD) are primarily validated based on observed differences between cognitively healthy elderly and AD patients [1–5].

Investigating biomarkers in subjects with preclinical AD (AD biomarker positive but cognitively normal) is important as clinical trials of new drugs shift to disease prevention in the still cognitively normal elderly [6, 7]. Biomarker changes may present itself as early as 20 years prior to disease onset and therefore early intervention is important [8]. Selecting subjects with preclinical AD for clinical trials may aid in demonstrating modification of disease progression due to treatment with drugs targeting core pathophysiological processes and treatment of patients with preclinical AD may ultimately prevent conversion to AD. Characterization of individuals with preclinical AD by identifying biomarkers indicative of the earliest pathophysiological processes involved in AD is therefore of the utmost importance. Preferably minimally invasive methods are used to identify AD pathology, especially in otherwise healthy subjects.

The accumulation of amyloid plaques and intracellular neurofibrillary tangles consisting of misfolded phosphorylated tau (Ptau) protein during the development of AD eventually leads to synaptic dysfunction after which axonal damage occurs and cognitive changes can be observed. While this protein-related process is ongoing, the immune system is also responsive [9].

Misfolded and aggregated proteins can bind to pattern recognition receptors on microglia and astroglia, and trigger an innate immune response characterized by release of inflammatory mediators, which contribute to disease progression and severity [10]. Differences in immune CSF biomarkers, such as YKL-40, MCP-1, and eotaxin-1 have been well established between healthy elderly and AD patients [11–15]. An observable neuroinflammatory response of the immune system to protein aggregation could mean that the process of neurodegeneration leading to AD has already started [9]. Measurement of these innate neuroimmune response-related biomarkers in the preclinical AD stages may help to predict which cognitively healthy elderly are more likely to develop AD.

YKL-40 (also known as chitinase-3-like protein-1 [CHI3L1]) is a glycoprotein, which is mainly expressed in astrocytes. AD patients have significantly higher YKL-40 levels in the CSF compared with healthy controls however it is not a specific biomarker for AD, because it merely reflects the inflammatory progress. YKL-40 is suitable as a marker for clinical drug trials to give information about neurodegeneration and glial activation independently of tau and Aβ [12]. Plasma YKL-40 levels have been investigated in patients with AD and in healthy elderly controls [15] but not yet in subjects with preclinical AD.

Glial fibrillary acidic protein (GFAP) is a marker for astrogliosis and has been reported to be increased postmortem in brains of patients with AD and in CSF of patients with AD [16, 17]. Verberk et al., (2021) found GFAP to be associated with in increased risk of dementia and a sleeper rate of cognitive decline and they conclude that GFAP has the potential to be a prognostic blood-based biomarker for AD in their cohort of cognitively normal older people [18]. Another recent study showed elevated plasma GFAP levels in subjects with preclinical AD which could mean that astrocytic damage or activation starts in the preclinical phase of AD [19].

Chemokines are a family of chemoattractants, which play a vital role in cell migration from blood into tissue and vice versa, and in the induction of cell movement in response to a chemical (chemokine) gradient by a process known as chemotaxis [20]. In addition, chemokines have recently been shown to have a function in the nervous system as neuromodulators. Two chemokines (monocyte chemoattractant protein-1 [MCP-1] and eotaxin-1) have previously been reported to be correlated with greater memory impairment in MCI and AD [11]. In a recent study, these chemokines were demonstrated to be able to discriminate between healthy subjects and subjects with MCI and AD [13].

In the current study, we aimed to investigate plasma biomarkers related to neuroinflammation associated with AD in a cohort of subjects with preclinical AD and to compare these to healthy elderly. Using a preclinical subject population will add valuable information to the body of literature on the onset of AD.

## Methods

This was an exploratory sub-study of a previously performed study registered in the international trial register with ID number: ISRCTN79036545. All study participants provided written consent for exploratory analyses of material obtained during study execution.

The main study was approved by the ethics committee of the Leiden University Medical Center (LUMC), the Netherlands. The study was conducted according to the Dutch act on Medical Research Involving Human Subjects (WMO) and in compliance with Good Clinical Practice (ICH-GCP) and the Declaration of Helsinki.

### Participants

Samples of 100 healthy male and female participants of 65 years of age and older were selected from the main study in health elderly [21]. All subjects were healthy volunteers without cognitive complaints who registered for participation voluntarily. Of these 100 subjects, 50 subjects were selected with a CSF Aβ1-42 profile consistent with Alzheimer’s disease and were classified as subjects with probable brain amyloidosis, referred to as preclinical AD. A healthy control group of 50 subjects was selected based on subjects having high levels of CSF Aβ1-42. Aβ1-42 was measured in CSF using the fully automated Elecsys platform [22] at the Neurochemistry Lab Amsterdam UMC, using in-house confirmed cutoffs [23]. Lowered Aβ levels classified as amyloid abnormal and consistent with the presence of Alzheimer pathology were dichotomized by creating a group of “Aβ positive subjects” (Aβ+ = < 1000 pg/ml) and “Aβ negative subjects” (Aβ− = > 1000 pg/ml). All the subjects visited Centre for Human Drug Research (CHDR) between October 2017 and November 2018. Main exclusion criteria were a diagnosis of a cognitive disorder (including but not limited to MCI, AD, Lewy Body dementia [LBD], frontotemporal dementia [FTD]), history of psychiatric disease in the past 3 years, Mini-Mental State Examination (MMSE) ≤ 24, Geriatric Depression Scale (GDS) ≥ 6, presence of drug or alcohol abuse (<2 standard drinks per day for female and <3 standard drinks per day for male), use of any medication that was expected to influence central nervous system function or is contraindicative of the performance of a lumbar puncture.

All subjects visited the clinical research unit once and underwent blood sampling at predefined time points (0, 2, and 4 h]). A single lumbar puncture was performed for the collection of CSF (at 4 h, either in the morning or afternoon). Furthermore, an automated CNS test battery was performed to collect data related to different domains of CNS functioning. The Clinical Dementia Rating scale (CDR) was assessed during the study day.

In the context of a post hoc analysis, subjects were also dichotomized based on the Ptau/Aβ1-42 ratio. Previous studies have shown that the use of ratio scores may be superior to the use of a single biomarker [24, 25]. Ptau information was known from the main study and determined by measuring Ptau in CSF using the fully automated Elecsys platform [22] at the Neurochemistry Lab Amsterdam UMC, using the Ptau/ Aβ1-42 ratio >0.02 cutoff for preclinical AD definition. Subjects with a score <0.02 were classified as healthy subjects.

### Blood sampling

Approximately 10mL of blood was collected via an i.v. catheter placed in an antecubital vein in the arm in appropriate K2EDTA tubes (BD, USA) at the predefined time points mentioned above. Following blood centrifugation within 1 h at 2000g for 10 min at 4°C, the plasma aliquots were divided into 0.5mL aliquots in Sarstedt polypropylene tubes and stored at −80°C. All blood samples for analyses of YKL-40, GFAP, MCP-1, and eotaxin-1 are collected in a non-fasted state within 1 h of collection of the CSF sample

### Lumbar puncture

Lumbar punctures were performed by a trained physician with a 25G atraumatic lumbar puncture needle (Braun, 25G). The needle was placed at the L3–L4 or L4–L5 interspace with the subject in supine or sitting position. 4 ml CSF was collected in a 15 mL polypropylene tube (Corning, USA). CSF was centrifuged within 1 h, at 2000g for 10 min at 4°C, and stored at −80°C [26].

### Apolipoprotein E genotyping

Apolipoprotein E (APOE) genotyping was performed after isolating DNA from EDTA blood by the laboratory of human genetics (department of human genetics and endocrinology, Leiden University Medical Center LUMC). DNA was isolated using a QIAamp DNA Blood MINI kit after which a polymerase chain reaction (PCR) technique was applied on the clean DNA. A sequential analysis (according to the Sanger method) then determined the APOE genotype. One or 2 APOE ε4 alleles classified subjects as APOE ε4 carriers, when no APOE ε4 alleles were present a subject was classified as noncarrier.

### Measurement of YKL-40, GFAP, MCP-1, and eotaxin-1

YKL-40 (Chitinase 3-like 1 [CHI3L1]) was measured in the plasma samples using the CHI3L1 Human ELISA Kit (Thermo Fisher) according to the manufacturer’s instructions. YKL-40 was measured previously in a larger sample and not for the sole purpose of this study [21]. Results of the 100 subjects selected for this study have been used in the analyses.

Plasma GFAP concentrations were measured at Amsterdam University Medical Centers (Amsterdam UMC) using the Simoa GFAP Discovery kit on the Single molecule array (Simoa) platform (Quanterix, Billerica, USA). MCP-1 and Eotaxin-1 were also measured at the Amsterdam UMC using Meso scale discovery (MSD, Rockville, MD, USA) assays according to the kit instructions.

### Statistical methodology

Visual checks on the ranges of biomarker and clinical characteristic test scores for each group based on CSF amyloid beta status were done using scatter plots, as well as Tukey boxplots. Independent *T*-test, Pearson chi-square test, and Mann-Whitney tests were applied as appropriate.

To establish differences between subject groups in biomarkers, data is analyzed using an ANCOVA, where age, sex, and E4 status are added to the model as covariates. After including all covariates, the analysis was repeated with only the significant covariates added to the model. Variables were Log transformed where applicable. Least square means were calculated for all 4 inflammatory biomarkers using both the Aβ+/Aβ− cutoff and Ptau/Aβ1-42 ratio. All analyses were carried out using SAS for Windows V9.4 (SAS Institute, Inc., Cary, NC, USA). A *p*-value of <0.05 was considered significant.

## Results

### Demographic and clinical characteristics

The mean age of the total group of study participants (*n*=100) was 72.6 (4.6) years old with 62 male and 38 female subjects. Mean overall MMSE score was 28.7 (0.49) and 32 subjects were APOE ε4 carriers. All subjects had a CDR score of 0.

### Comparison of plasma YKL-40, GFAP, MCP-1, and eotaxin-1 between Aβ+ and Aβ− subjects

**Fig. 1:**
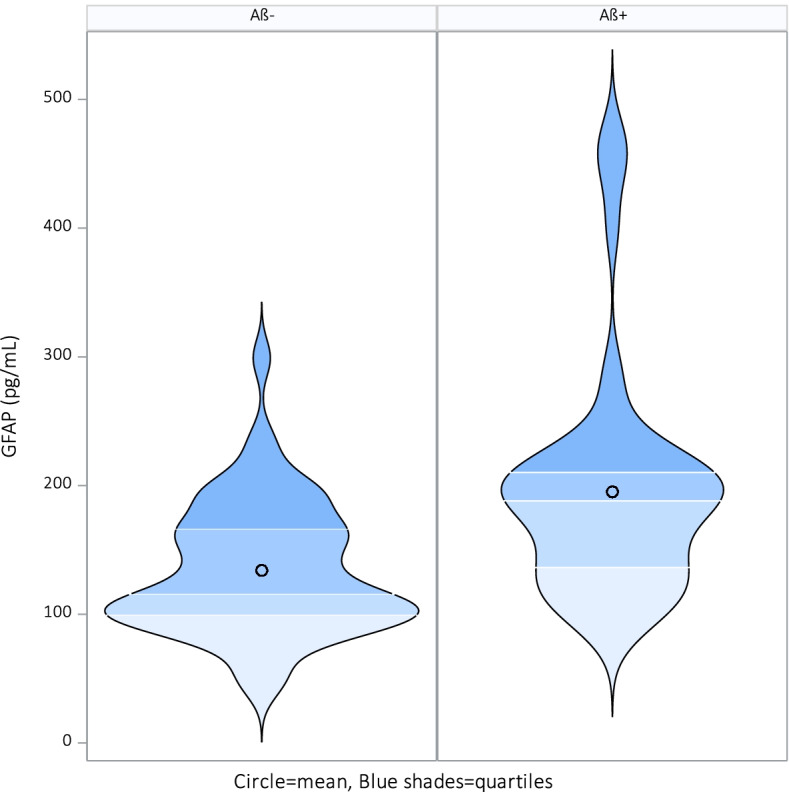
Significant violin plot for GFAP among healthy elderly subjects with a CSF profile consistent with Alzheimer’s disease, *n*=50 (Aβ+ [CSF Aβ42 <1000] versus healthy elderly subjects with normal CSF Aβ−, *n*=50 [CSF Aβ42>1000])

Table 1 presents the cross-sectional demographics and clinical characteristics of the studied population based on Aβ+/Aβ− groups. The APOE ε4 status was significantly different between Aβ+ and Aβ− subjects. All other clinical characteristics do not differ significantly between the Aβ+ and Aβ− groups. Plasma GFAP concentration was significantly higher in the Aβ+ group compared to the Aβ− group before and after adjusting for covariates age and sex, variables that also correlated significantly with GFAP, see Fig. 1. YKL-40, MCP-1, and eotaxin-1 were not significantly different between the Aβ+ and Aβ− group None of the biomarkers correlated with the MMSE score.

**Table 1:** Cross-sectional demographics and clinical characteristics of the studied population based on Aβ+/Aβ− groups

	Aβ+ (***n***=50)	Aβ− (***n***=50)	***p***
Aβ level (mean, SD)	706.0 (174.36)	>1700	
Sex (male/female)	33/17	29/21	0.41
BMI (mean, SD)	26.07 (3.95)	25.17 (3.44)	0.225
Age (years, mean, SD)	73.40 (4.72)	71.88 (4.45)	0.101
APOE ε4 carrier (*n*, %)	25 (50%)	7 (14.6%)	**0.003**
MMSE (mean, SD)	28.60 (1.41)	28.82 (1.37)	0.431
CDR (mean, SD)	0 (0)	0 (0)	
GFAP pg/mL (mean, SD)	*N*=50 195.1 ± 87.13	*N*=50 134.0 ± 50.71	**<0.001**
YKL-40 pg/mL (mean, SD)	*N*=49 54,662.3 ± 39,697.31	*N*=49 82,947.1 ± 83,418.38	0.397
MCP-1 pg/mL (mean, SD)	*N*=50 91.74 ± 16.72	*N*=50 97.98 ± 34.01	0.358
Eotaxin-1 pg/mL (mean, SD)	*N*=50 195.0 ± 57.87	*N*=50 204.0 ± 94.80	0.783

*P* values in bold font were considered significant (*p*<0.05). Independent *T*-test and Pearson chi-square test were applied as appropriate

### Comparison of plasma YKL-40, GFAP, MCP-1, and eotaxin-1 between subjects divided based on Ptau/Aβ42 ratio

**Fig. 2:**
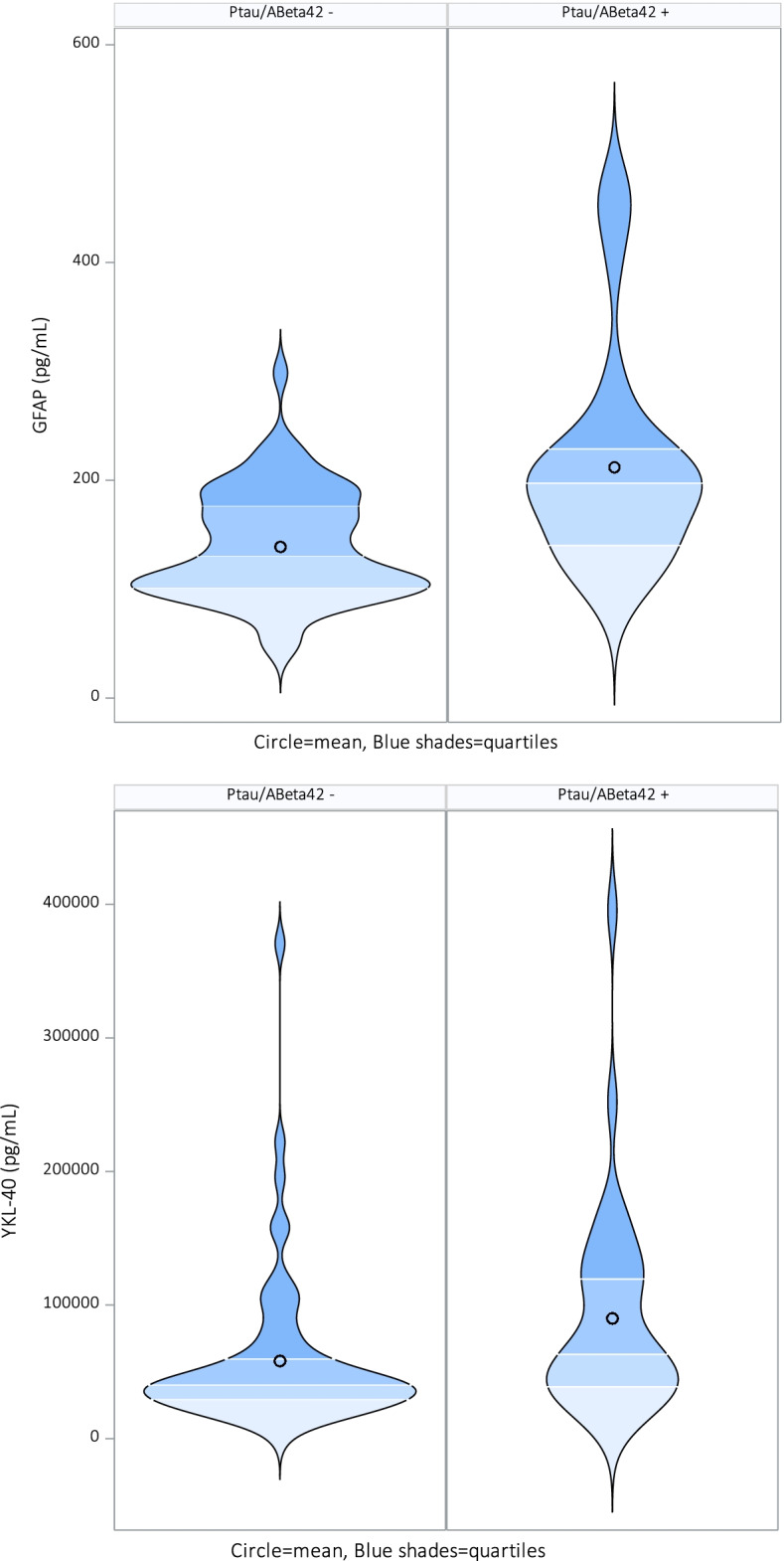
Significant violin plots for GFAP and YKL-40 compared to Ptau/Aβ42 ratio

Table 2 presents the cross-sectional demographics and clinical characteristics of the studied population based on the Ptau/Aβ1-42 ratio score. The APOE ε4 status was significantly different between the two groups divided by Ptau/Aβ1-42 ratio score. All other clinical characteristics do not differ significantly in groups. Plasma GFAP and plasma YKL-40 concentration were significantly higher in the preclinical AD group based on the Ptau/Aβ1-42 ratio before and after adjusting for covariates age, sex, and APOE ε4 status as these variables also correlated with GFAP, see Fig. 2. YKL-40 was significantly different between APOE ε4 carriers versus non-carriers. Eotaxin-1 was significantly different between the sexes. MCP-1 did not show any difference.

**Table 2:** Cross-sectional demographics and clinical characteristics of the studied population based on the Ptau/Aβ1-42 ratio score

	Ptau/Aβ + (***n***=36)	Ptau/Aβ – (***n***=64)	***p***
Ptau/Abeta42 ratio	0.04 (0.012)	0.01 (0.003)	
Aβ level (mean, SD)	685.2 (163.7)	1494.2 (401.9)	
Sex (male/female)	26/10	36/28	0.166
BMI (mean, SD)	26.2 (3.8)	25.3 (3.7)	0.338
Age (years, mean, SD	73.8 (4.9)	72.0 (4.4)	0.039
APOE ε4 carrier (*n*, %)	18 (50%)	14 (22.6%)	**0.001**
MMSE (mean, SD)	28.5 (1.5)	28.8 (1.4)	0.314
CDR (mean, SD)	0 (0)	0 (0)	
GFAP pg/mL (mean, SD)	211.8 ± 97.6	138.8 ± 49.9	**<0.001**
YKL-40 pg/mL (mean, SD)	*N*=38 87038.7 ± 74252.3	*N*=145 60583.7 ± 54067.1	**0.012**
MCP-1 pg/mL (mean, SD)	*N*=34 92.6 ± 18.4	*N*=64 96.4 ± 30.7	0.602
Eotaxin-1 pg/mL (mean, SD)	*N*=34 193.6 ± 62.9	*N*=64 202.5 ± 86.7	0.630

*P* values in bold font were considered significant (*p*<0.05). Independent *T*-test, Pearson chi-square test, and Mann-Whitney tests were applied as appropriate

### Correlation between biomarkers

**Fig. 3:**
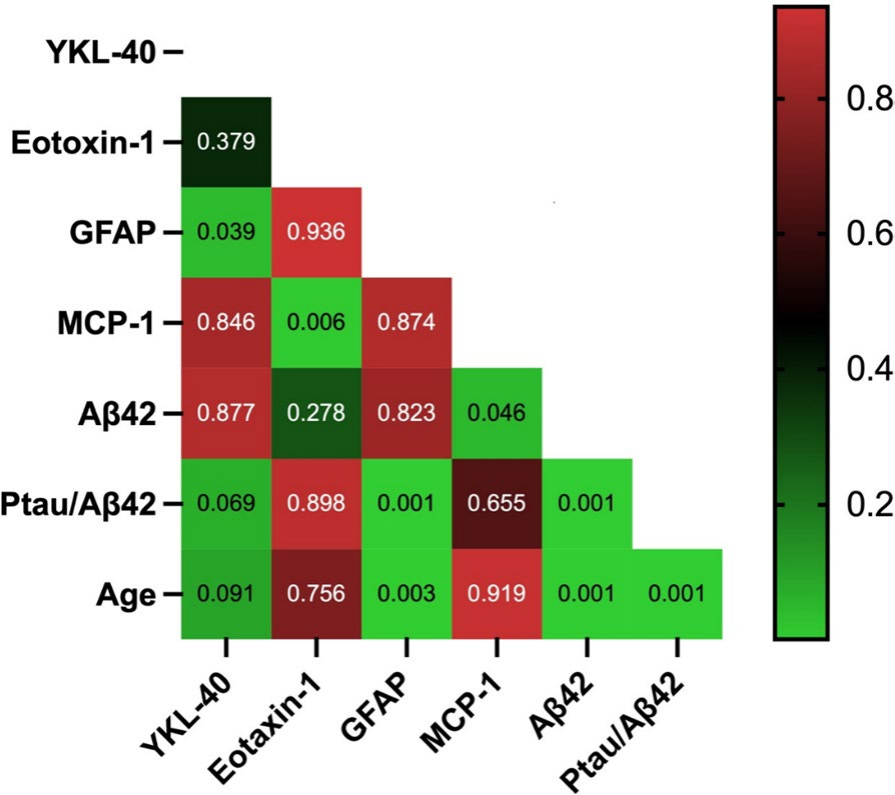
Heatmap *p*-values for biomarkers correlations YKL-40, GFAP, eotaxin-1, MCP-1, Aβ42, and Ptau/Aβ42

Figure 3 represents a heatmap with p-values calculated for all inflammatory biomarkers plus Aβ42, Ptau/Aβ42 ratio, and age. Plasma YKL-40, GFAP, Aβ42, and Ptau/Aβ42 ratio correlated with age. YKL-40 also correlated with GFAP and Ptau/Aβ42 ratio. GFAP correlated with Ptau/Aβ42 ratio. MCP-1 is positively correlated with eotaxin-1 and Aβ42. Aβ42 and Ptau/Aβ42 ratio are strongly correlated. *N*=121 for Aβ, which are the samples of all original subjects included in the main study except the subjects with a CSF Aβ42 concentration of >1700 as no exact concentrations are available.

## Discussion

In the current exploratory study, we aimed to investigate plasma biomarkers related to neuroinflammation associated with AD in a cohort of subjects with preclinical AD and to compare these to healthy elderly, both defined by Aβ1-42 CSF status. Of the four inflammatory plasma biomarkers investigated in this study, only GFAP was significantly higher in subjects with preclinical AD compared to healthy elderly. When defining preclinical AD based on the Ptau181/Aβ1-42 ratio, GFAP and YKL-40 were significantly different between groups. This could indicate that GFAP and YKL-40 are more sensitive markers of the incipient inflammatory process that occurs in response to the beta-amyloid misfolding and aggregation that is ongoing as indicated by the lowered Aβ1-42 protein levels in the CSF.

With the increasing prevalence of AD [27], it would be interesting to look at “biomarker-positive” subjects, 50% of whom will develop AD [24], and further investigate the course over time of the inflammatory biomarkers described here. As we found in the current study, evidence of astrogliosis as demonstrated by elevated GFAP was already increased in healthy subjects positive for CSF Aβ1-42. If we can further characterize these subjects, we may be able to define a group of healthy subjects more likely to develop AD and treat these subjects in early (neuroinflammatory or CSF Aβ1-42 lowering) clinical trials. Measurement of GFAP and YKL-40 in plasma is useful in healthy subjects with preclinical AD as it allows to determine the level of neuroinflammation in subjects possibly developing AD and can provide more information on the relationship between neuroinflammation and the development of AD. Disease-modifying treatments targeting neuro-inflammation early in the preclinical disease process of AD may delay disease progression and prevent or delay cognitive decline as inflammation can be expected to influence cognitive performance independently from Aβ pathology [28].

Our results showing an increase in GFAP in the preclinical stage are in line with Verberk et al., (2021) who studied a similar population of cognitively healthy elderly and found GFAP to be associated with increased risk of progression to dementia and steeper cognitive decline [18]. Aβ measured in plasma by Chatterjee et al. (2021) [19] in cognitively normal older adults resulted in two groups, Aβ+ and Aβ− subjects comparable to our studied population. This study also found that GFAP was elevated in subjects with preclinical AD. Our study therefore reproduces these study results, demonstrating that these findings are real and independent of the specific samples used by Chatterjee or by us. Pereira et al. (2021 )[28] mention that plasma GFAP might be specific to AD as it correlated with Aβ pathology in their study with comparable cognitive normal subjects, which is supported by the differences between groups found in our study but not the correlation with Aβ itself. Alternatively, this could be the result of a smaller sample size. Further research is needed to determine if GFAP can be used as a CSF-independent marker for (preclinical) AD.

When YKL-40 is measured in CSF, this could indicate that microglial activation is taking place, even though YKL-40 concentrations are already measurable in subjects without lowered Aβ measured in CSF [29]. Several associations have been found between CSF YKL-40 and neurodegenerative biomarkers in CSF namely total tau protein and significant differences have been found between AD patients, healthy elderly, and subjects with preclinical AD [30]. Demonstrating differences in plasma levels of YKL-40 between healthy elderly and subjects with preclinical AD could help to identify inflammatory processes in a less invasive manner. In our study, plasma YKL-40 did not correlate with CSF Aβ1-42 and was not different between subjects with preclinical AD and healthy controls. Thus, no conclusion can be drawn about glial activation by YKL-40 in response to the accumulation of Aβ in this particular sample of healthy subjects, perhaps because it is too early in the disease process to identify differences in YKL-40 concentrations in plasma. When redefining the subjects based on CSF Ptau181/Aβ1-42 ratio scores, plasma YKL-40 concentration was found to differ between groups. This comparison was performed post hoc, however. As plasma YKL-40 was not previously reported to be different between subjects with preclinical AD and healthy controls, this finding is of interest and a reason to further investigate this and confirm it in a properly powered study aimed at replication. Comparable to GFAP, YKL-40 levels increase with age, in CSF, and also in plasma. When measured in plasma, higher plasma YKL-40 concentrations seem to be correlated with male sex, older age, APOE ε4 status, and cerebral accumulation of Aβ measured with PET [31]. Our sample did not find YKL-40 to be correlated with age, sex, APOE ε4 status and Aβ measured in CSF. GFAP showed to be correlated with sex, age, and Aβ status in our sample. GFAP and YKL-40 can be found in a vast range of peripheral cells expressing it and might therefore be measurable in plasma. Previous studies, however, conclude that measuring GFAP in plasma is related to CNS inflammation and severity of disease [32, 33]. YKL-40 has been found to be increased in subjects with streptococcal pneumonia and could therefore have a peripheral origin and confound to the measurability in plasma which should be taken into account when interpreting YKL-40 results in plasma [34].

The subjects investigated in the current study were part of a larger observational study; therefore, information on cognitive status was measured using a computerized cognitive test battery and several paper and pencil tasks were available. Our two groups, preclinical AD and healthy elderly, were specifically different regarding Abeta1-42 measured in CSF. We divided GFAP and YKL-40 scores into “high” levels and “low” levels of inflammation by using the median and compared these groups with the total group of subjects. None of the cognitive domains (e.g., memory, attention, overall cognitive performance measured with MMSE and CDR) differed significantly between groups and therefore there was no indication of early cognitive decline in the otherwise healthy subjects with elevated neuroinflammatory markers. This is in contrast to other, longitudinal studies, which have found that plasma (and CSF) GFAP could predict global cognitive decline [18] even though plasma GFAP was not always measured longitudinally [28].

## Limitations

The correlation that we found between CSF Aβ42 and CSF Ptau/Aβ42 ratio is inherently based on the use of CSF Aβ42 in the latter ratio. YKL-40 being significantly different in the Ptau/Aβ42 ratio condition and not being different based on Aβ alone could be a result of this.

GFAP correlated significantly with CSF Ptau/Aβ42 ratio but not with CSF Aβ42, which can possibly be explained by differences in sample size. The calculation of GFAP in correlation with Aβ only includes the Aβ+ subjects as these are continuous values (*n*=50), the Aβ− subjects all had Aβ levels of >1700 (no exact value). For calculating the ratio score Ptau/Aβ42 the whole data set could be used (n=98) with Aβ− =1700. When including a larger N, also including the 50 Aβ− subjects, the correlation between GFAP and CSF Aβ42 could have been significant as we have established a difference between groups on GFAP and Aβ+/Aβ− subjects. For the calculation of the correlation between Aβ42 and age, the original data set of 200 subjects was used, of which 121 subjects had exact Aβ42 values. The subjects with Aβ42 concentrations of >1700 pg/mL were not included as no exact concentrations were known; it was only indicated that levels were >1700 pg/mL. It is, however, unlikely that the correlation found between Aβ42, and age would be non-significant if exact values for all subjects with levels >1700 pg/mL were available.

The subjects included in this study were not referred to a memory clinic but voluntarily participated in this study. No subjects with proof of (subjective) memory complaints participated, demonstrated by a MMSE of >24 during prescreening, and during the study confirmed by a CDR of 0 and IADL of 0. However, subjects with insecurities about their cognitive performance might be more likely to participate in observational studies.

This study was exploratory and further research is needed to confirm the results. Data in this study was not corrected for multiple comparisons.

## Conclusions

Measuring GFAP and YKL-40 in plasma of subjects with preclinical AD could be of added value to further differentiate subjects with lowered CSF Aβ42 from otherwise healthy elderly to better define the preclinical AD status. However, this study was cross-sectional and subject discrimination needs further analyses. If further research shows that these inflammatory plasma biomarkers are specific for (preclinical) AD, measuring these can be an important step forward in characterizing otherwise healthy elderly with preclinical AD in a less invasive manner.

## Data Availability

The datasets generated during this study are not available by request.

## Abbreviations

Aβ: Amyloid beta
AD: Alzheimer’s disease
AUC: Area under the curve
CHI2L1: Chitinase-3-like protein-1
CI: Confidence interval
CSF: cerebrospinal fluid
FTD: Frontotemporal dementia
GFAP: Glial fibrillary acidic protein
LBD: Lewy body dementia
MCI: Mild cognitive impairment
MCP-1: Monocyte chemoattractant protein-1
MMSE: Mini-Mental State Examination
PD: Parkinson’s disease
PET: Positron emissive tomography
Ptau: Phosphorylated Tau
ROC: Receiver operating characteristics
WHO: World Health Organization
